# Status of association studies linking diabetes mellitus and periodontal disease in India

**DOI:** 10.4103/0973-3930.62595

**Published:** 2010

**Authors:** Anirudh B. Acharya, Aparna Satyanarayan, Srinath L. Thakur

**Affiliations:** Department of Periodontics, S.D.M. College of Dental Sciences and Hospital, Dhavalnagar, Dharwad - 580 009, Karnataka, India

**Keywords:** Asian Indians, diabetes mellitus, India, periodontal disease, periodontitis

## Abstract

**Background:**

The prevalence of diabetes mellitus (DM) and periodontal disease / periodontitis (PD) is high, and the association of these two as risk factors influencing each other has been recognized and is extensively documented. However, a majority of these association studies have been done in developed countries and / or in developing countries other than India.

**Objective:**

To review, quantify and qualify the status of the published indexed scientific literature regarding the bidirectional association of DM and PD in India.

**Method:**

An internet search of the literature was conducted to examine relevant association studies of DM and PD in India. As a matter of interest, the related articles were searched manually from one non-indexed national (India) publication of periodontology until October 2008. Specific focus was on indexed literature and only these were considered for the review.

**Result:**

Eleven pertinent indexed publications addressing the concern with regard to population in India were identified. Most of them were in agreement with the accepted mechanisms involving these two diseases, but more supported a unidirectional view of interaction, that is, advocating the role of DM in a worsened periodontal condition. One study with an opposite view was recognised.

**Conclusion:**

The paucity of such research in India, which will be a hub of diabetics in the near future, reflects the need to make available a strong body of evidence in the indexed literature relating not only to DM and its ‘sixth complication’, PD, but also the strong influence of PD on DM.

## Introduction

A PubMed and a general internet search was carried out to identify the relevant indexed scientific publications, specifically addressing the association of diabetes mellitus (DM) and periodontal disease / periodontitis (PD) involving the population in India. Considering the importance of the link between these two diseases, a manual search was also undertaken of one non-indexed national (India) publication (of the Indian Society of Periodontology) until October 2008. The latter was not considered in the review.

## Overview

The prevalence of DM for all age groups worldwide was estimated to be 2.8% in 2000 and an anticipated 4.4% in 2030. The total number of people in the world with diabetes is projected to rise from 171 million in 2000 to 366 million in 2030. India ranked number one as the country with the highest number of diabetes patients in 1995, at 31.7 million in 2000, with a projected 57.2 million in 2025, and 79.4 million in 2030, retaining its top position. To a greater extent Asian Indians have a racial predisposition and other unique risk factors to develop DM. DM has acquired a pandemic status in India.[1–5]

Research in several countries indicates that 5 – 20% of any population will have severe PD and a majority of adults suffer from moderate forms of this disease.[6] Evidence suggests that one out of two adults above the age of 35 years have PD in India, and 35% of the teeth extracted are as a consequence of PD.[7] One study reported 13.3, 12.5, 29.1, and 97% prevalence of various periodontal parameters.[8]

The increased prevalence and severity of PD typically seen in patients with DM, especially those with poor metabolic control, has led to the designation of PD as the ‘sixth complication of diabetes’.[9] The American Diabetic Association has officially recognized that PD is common in patients with diabetes, and the Association's Standards of Care include taking a history of current or past dental infections as part of the medical examination.[10,11]

## Diabetes mellitus influencing periodontal disease

Diabetes is a risk factor for PD, and the level of glycemic control is an important determinant in this relationship.[12,13] An increase in the extent and severity of PD in diabetics have been reported by various investigators,[14–17] although some have not found a significant relationship between diabetes and PD.[18–20] Early research focused on the differences in the pathogenic microflora in subjects with diabetes as a probable explanation for increased PD. However, studies have shown that there is no difference between the microbiota in diabetic and non-diabetic individuals.[21,22]

### The influence of DM on PD could be the due to:

1. A direct causal relationship in which DM acts as a modifier of PD. The advanced glycation end products (AGEs) formed as a result of hyperglycaemia / hyperlipidemia can act in two ways – by binding to receptors, which can transform macrophages to produce proinflammatory cytokines such as interleukin-1 (IL-1), IL-6, and tumour necrosis factor-α (TNF-α). Formation of AGEs results in collagen accumulation in the periodontal capillary basement membrane, causing membrane thickening.[23] This increases the thickness of the vessel walls and decreases tissue perfusion and oxygenation. These morphological changes could be responsible for the increased susceptibility to infections, vascular changes, and impaired healing, commonly associated with diabetics. The increase in proinflammatory cytokines in the periodontal tissues could also explain the increased tissue destruction.
2. The less accepted hypothesis is that of a common pathological defect that makes the host more susceptible to either or both of these diseases. Among the common pathways, genetic and immunological mechanisms have been extensively studied. Studies by Kornman *et al*.,[24] have shown an association between Human Lymphocyte -A (HLA) antigens, particularly the HLA-DR4 gene and PD. Type 1 diabetics have been seen to express either the HLA –DR3 or DR4 configuration. This genetic association could result in a host susceptible to both PD and diabetes.
3. Immunological mechanisms: Both type 1 and 2 DM as well as PD can be considered to be maladapted or upregulated responses of the immune system to environmental stressors. The stressors for PD would be bacterial plaque, tobacco smoking, and psychological stress, and for type 1 DM, bacteria, viruses, and emotional stress. Overeating and physical stress would be the stressors for type 2 DM. These environmental factors will result in an increased inflammatory response, which includes macrophages / monocytes, lymphocytes, and endothelial cells, thus altering the host response.

It is possible, however, that these proposed mechanisms are not independent and can function together to result in a complicated set of events.

## Periodontal disease influencing diabetes mellitus

Periodontal disease increases the risk of poor glycemic control and other diabetic complications,[25,26] and studies have shown improved glycemic control with reduction in PD.[27–29] Noteworthy improvements in glycemic control have been observed after treatment and reduction of PD.[30,31]

Periodontal diseases may induce or perpetuate an elevated systemic chronic inflammatory state[32] and may also result in increased insulin resistance and poor glycemic control.[33] Treatment that reduces periodontal inflammation may restore insulin sensitivity, resulting in improved metabolic control. It is possible that PD may serve as initiators or propagators of insulin resistance in a way similar to obesity, thereby, aggravating glycemic control. A significant association between obesity and PD has been established.[34] Adipocytes are believed to increase the blood levels of TNF-α. Monocytes from patients with DM produce 24 – 32 times the level of TNF-α when stimulated by periodontal pathogens than do monocytes from subjects without diabetes.[35] TNF-α enhances insulin resistance. Studies by Nishimura *et al.*,[36] suggest that chronic periodontal infection contributes to insulin resistance. Chronic upregulation of TNF-α in response to lipopolysaccharide (LPS) and other cell surface toxins from bacteria in the subgingival biofilm (dental plaque) is probably a mechanism contributing to a state of insulin resistance in individuals at risk.

Association between PD and diabetes has always been controversial. There have been studies indicating a higher prevalence of PD in diabetic patients compared to healthy subjects and this had led to the unidirectional view that DM increased PD. However, a United States National Health and Nutritional Examination Survey (NHANES-III) data in 1996,[37] confirmed the bidirectional relationship between DM and PD. The analysis showed that the prevalence of DM in patients with PD is double that seen in non-periodontitis patients and there was higher prevalence of PD in diabetics compared to non-diabetics. The two-way relationship between DM and PD has been recognized.[38]

The global risk factors for type 2 DM have been identified.[39] The specific risk factors identified for the increased prevalence of DM in India are central obesity, tuberculosis, any other recurrent infection or ulcer, premature atherosclerosis, stress hyperglycemia, drugs causing hyperglycemia, and low birth weight due to intrauterine malnutrition.[40] The uniqueness of the genetic predisposition of Asian Indians to DM have also been documented.[41]

## The association studies in India

The salient features of the studies of DM and PD undertaken in India are put forth.

Circulating immune complexes were found to be significantly high in patients with type 2 diabetes and non-diabetic patients with PD as compared to controls. The circulating immune complexes were significantly higher in the diabetics as compared to the non-diabetics, suggesting a role in the pathogenesis of PD in diabetic patients.[42]

In another study, cell-mediated and humoral immune responses were assessed in 50 type 2 diabetic patients and 50 non-diabetic patients with PD by enumerating the total and high affinity rosette forming cells (for cell-mediated response), and estimating serum immunoglobulins G, A, M, D, and E {IgG, IgA, IgM, and IgE (for humoral response)}, respectively. The results were in concurrence with Zambon *et al.*[43] and Ranney *et al.*,[44] indicating significant elevations in the humoral immune profile of diabetic and non-diabetic patients with PD as compared to controls. No significant changes were observed in the cell-mediated humoral response. The investigators opined that the defective host response in diabetics was predisposing to the development of PD as compared to non-diabetics.[45]

The total hemolytic complement activity and its fractions, C3 and C4, were determined in 50 type 2 diabetic and non-diabetic patients with PD using the modified method of Mayer and a radial immunodiffusion technique, respectively. The values were compared with the controls. An elevation of total hemolytic complement activity was observed in both diabetic and non-diabetic patients, compared to controls. The diabetic patients with PD showed a significantly higher complement activity compared to non-diabetic patients with PD. The C3 and C4 values were significantly elevated in both diabetic and non-diabetic patients when compared with the control group. The elevation was more pronounced in the diabetic groups. The increased incidence of PD in diabetics was probably due to the increased susceptibility of diabetic patients to infections, which altered the immune response.[46]

Estimation of the concentration of salivary immunoglobulins G, A, and M (IgG, IgA, and IgM) in the saliva of diabetic and non-diabetic patients with PD revealed a non-significant increase of IgM in diabetic patients. IgG and IgA were found to be significantly increased in diabetic patients with PD, compared to the non-diabetic patients and controls. The increased incidence of PD in diabetics was attributed to the antigenic challenge and an altered immune response.[47]

Gingival tissue samples were assessed for immunoglobulins G, A, and M (IgG, IgA, and IgM) from diabetic and non-diabetic patients with PD. The levels of IgG and IgA were higher in diabetic and non-diabetic patients with chronic PD as compared to healthy subjects, with a significantly higher level of the same immunoglobulins in diabetics as compared to non-diabetics with PD.[48]

Sixty-two uncontrolled diabetic and 60 non-diabetic patients between the ages of 35 and 66 years were examined and dental plaque and calculus, and gingival and periodontal indices were studied. Correlation of salivary calcium and blood sugar with periodontal health and dental calculus deposition was also carried out. It was shown that there was an increase in PD status duration in uncontrolled diabetic patients. It was also observed that salivary calcium level was significantly higher in uncontrolled diabetics, aiding in dental calculus formation, and hence, an increase in the severity of PD.[49]

The expression of matrix metalloproteinases -8 and -9 in gingival tissue extracts from diabetic and non-diabetic patients with chronic PD and from healthy individuals were measured using gelatin zymography and western blotting techniques. The matrix metalloproteinases were significantly increased in diabetic patients with chronic PD as compared to non-diabetic patients with chronic PD. Expression of these enzymes may contribute to the altered healing mechanism due to the diabetic condition. It was suggested that treatment strategies aimed at inhibition of these matrix metalloproteinases could result in better healing of chronic PD, which is aggravated in diabetic patients.[50]

Vinitha *et al*.,[51] evaluated the dental status of 704 Asian Indian diabetic patients, which revealed a high prevalence of PD. They observed 87.2% with PD and 52.1% with advanced PD, reflected by mobility of teeth. It is noteworthy that this study was undertaken owing to the lack of data regarding PD in Asian Indian diabetics.

Shetty *et al*.,[52] in a comparison of neutrophil functions in diabetic and healthy subjects revealed defects in neutrophil functions in diabetic individuals as measured by chemotaxis, phagocytosis, microbicidal function, and super oxide release, which was in agreement with studies done by Alastair *et al*.,[53] Bybee *et al*.,[54] and Walters *et al*.[55] This study concluded that impaired neutrophil chemotaxis, defective phagocytosis of *Porphyromonas gingivalis (an important periodontal pathogen)* by neutrophils, and the intracellular killing capacity of neutrophils was reduced in diabetic patients and the super oxide released by diabetic neutrophils was drastically increased. Hence, these mechanisms could make a diabetic patient more susceptible to PD.

The levels of β-glucuronidase in the gingival crevicular fluid (GCF-an inflammatory exudate seeping from the gingival sulcus surrounding each tooth) in diabetics and non-diabetics with chronic PD and also in controls, was estimated using spectrophotometric analysis. PD patients expressed more severe periodontal destruction with increased β-glucuronidase, irrespective of their diabetic status. It was demonstrated that there was a higher level of GCF β-glucuronidase activity in patients of DM with chronic PD, as compared to non-diabetics with chronic PD. The findings were in agreement with Bang *et al*.,[56] Lamster *et al*.,[57] and Bacic *et al.*[14] It was observed that diabetics were at a higher risk for PD as reported by Chowdhary *et al.*[58]

A randomized controlled clinical trial by Singh *et al*.,[59] involving 45 type 2 diabetes patients, showed that periodontal therapy had a role to play in improved glycemic control, moreso, in those patients who were subjected to periodontal treatment and adjunctive doxycycline (100 mg daily for 14 days). This was compared with controls who did not receive any periodontal intervention.

## Discussion

From a historical perspective, as early as 1889, Grunert {reference not furnished} reported periodontal changes in diabetics and it was Williams (1928), who described ‘diabetic periodontoclasia’.[60]

Diabetes mellitus and PD are closely linked chronic diseases with similarities in pathobiology, and inflammation is the central player in this association. Mounting evidence demonstrates that diabetes is a risk factor for periodontitis and possibly oral premalignancies and oral cancer. The systemic inflammatory response generated by inflamed periodontal tissue may in turn exacerbate diabetes, worsen cardiovascular outcomes, and increase mortality.[61,62]

An appraisal of the indexed scientific literature resulted in a miniscule corpus of evidence regarding the association of DM and PD, in India. Although well-documented and evidenced in recent times, the association of these two disease entities with a bidirectional mechanism influencing each other has not reached a consensus level in India, pertaining to its population.

Scrutiny of the collected data clearly indicates the alterations in immunological, pathological, and biochemical responses exhibited by diabetic patients, which have a role in aggravating PD severity.[42,45–48,50,52,58] These are in conformity with the contemporary views that diabetics are at a higher risk for PD. The randomized controlled clinical trial by Singh *et al*.,[59] reflected the opposing view of the influence PD had on the severity of DM, which was in compliance with Grossi *et al*., Iwamoto *et al*., and Tervonen *et al*.[63–65]

The clarity of these studies emphasize the bidirectional mechanisms in play between DM and PD in the population of India. However, two aspects are to be noted. One that more research needs to be done in India in this field, and two, a consensus needs to be arrived at, specifically with regard to the effect of PD on DM, considering the unique predisposition of Asian Indians' susceptibility to DM.

A manual search of the bulletins and journals of the Indian Society of Periodontology from 1994 to October 2008 (after which the journal was indexed as a scientific publication) was done. This particular publication was chosen for the obvious reason that this has been the frontrunner among the dedicated researches in the field of periodontology in India. Although an array of numerous studies was documented in this publication addressing the matter regarding the association of DM and PD in India, citation concerns excluded the possibility of including them in this review. It is opined that these studies would be of good value to the scientific literature, especially considering the lack of such research in the general population of India. Without a doubt, the requirement of a sound base of knowledge directed toward this topic is justified, owing to the pandemic proportions DM is expected to attain in India in the near future.

Association studies are of great significance. They allow testing the consistency, strength and specificity of associations to measure the risk aspects of disease, and also to assess the degree of exposure and biologic plausibility, among other factors to verify the relationship between two entities.[66–69]

## Conclusion

The link between DM and PD is well known. Each has a specific influence on the other independently or in a more complicated and less understood pathobiological mechanism. Considering the high prevalence of both these diseases in India, a substantial volume of data needs to be constructed by way of concerted research between the medical and dental fraternities, to enable management of these conditions in the population of India. A database of indexed publications addressing this subject is of high priority.
